# Induced Neurocysticercosis in Rhesus Monkeys (*Macaca mulatta*) Produces Clinical Signs and Lesions Similar to Natural Disease in Man

**DOI:** 10.1155/2014/248049

**Published:** 2014-12-17

**Authors:** N. Chowdhury, A. Saleque, N. K. Sood, L. D. Singla

**Affiliations:** ^1^Department of Veterinary Parasitology, College of Veterinary Science, Guru Angad Dev Veterinary and Animal Sciences University, Ludhiana 141004, India; ^2^Department of Veterinary Parasitology, College of Veterinary Science, Assam Agricultural University, Guwahati 781022, India; ^3^Department of Teaching Veterinary Clinical Complex, College of Veterinary Science, Guru Angad Dev Veterinary and Animal Sciences University, Ludhiana 141004, India

## Abstract

Neurocysticercosis is a serious endemic zoonosis resulting in increased cases of seizure and epilepsy in humans. The genesis of clinical manifestations of the disease through experimental animal models is poorly exploited. The monkeys may prove useful for the purpose due to their behavior and cognitive responses mimicking man. In this study, neurocysticercosis was induced in two rhesus monkeys each with 12,000 and 6,000 eggs, whereas three monkeys were given placebo. The monkeys given higher dose developed hyperexcitability, epileptic seizures, muscular tremors, digital cramps at 10 DPI, and finally paralysis of limbs, followed by death on 67 DPI, whereas the monkeys given lower dose showed delayed and milder clinical signs. On necropsy, all the infected monkeys showed numerous cysticerci in the brain. Histopathologically, heavily infected monkeys revealed liquefactive necrosis and formation of irregular cystic cavities lined by atrophied parenchymal septa with remnants of neuropil of the cerebrum. In contrast, the monkeys infected with lower dose showed formation of typical foreign body granulomas characterized by central liquefaction surrounded by chronic inflammatory response. It was concluded that the inflammatory and immune response exerted by the host against cysticerci, in turn, led to histopathological lesions and the resultant clinical signs thereof.

## 1. Introduction

Cysticercosis is an important zoonosis, endemic in many parts of the world, particularly in Latin America, Africa, Southeast Asia, India, China, and parts of Commonwealth of Independent States. It is relatively frequent in European countries, namely, Portugal, Spain, Poland, and Romania. It is also reported in North America, Australia, and New Zealand. However, neurocysticercosis is more serious and specific infection, caused by the ingestion of eggs of adult worm,* Taenia solium*, and consequent development of its larval stages, which localize in the central nervous system. The most common symptom of the disease in man is epileptic seizures, sometimes as high as 50–60%, in both the developed and developing countries [1–3]. Although some information is now available on the clinicopathological aspects of the condition in both man and animals [4–6], the precise knowledge of the mechanisms of its cerebral manifestations remains unclear. There are few reports of accidental ingestion of eggs and the subsequent development of the cysticerci in the central nervous system in nonhuman primates which may cause neurological signs and lesions similar to those in humans. Therefore, nonhuman primates may serve as an important experimental model for studying the clinical signs, pathology, and pathogenesis of neurocysticercosis particularly due to their behavior and cognitive responses. Hence, the present study was designed.

## 2. Materials and Methods

Seven rhesus monkeys of either sex, aged about 1-1/2-2-1/2 years, weighing 2.4–3.2 kg, procured from the State Zoo, were kept under critical observations in independent cages for a period of six months. The animals were maintained with pelleted monkey chow (Hindustan Lever Limited, India), soaked gram, and fresh fruits throughout the experimental period. Two of these animals were infected with 12,000 and 6,000 eggs [7–9], whereas three of these monkeys served as control. The morphology and viability of the proglottids (human origin) and recovered cysticerci from infected monkeys were studied both grossly and microscopically using flattened, fixed, and Borax carmine-stained specimens. The feeding cysticerci to two immunosuppressed hamsters produced immature worms, confirming the infectivity of the cysticerci. The number of eggs/oncospheres fed to each monkey was determined from 4-5 proglottids fixed in 10% formalin. These were macerated in pestle and mortar in 5–10 mL of formalin. The total number of oncospheres was estimated from the average of 10–15 drops of the fluid. Freshly shed proglottids were collected from the stool from persons from 2 to 3 villages located within 6-7 km from the university campus, brought to the laboratory in physiological saline, washed repeatedly to clean them, and processed in the same way as above to estimate average number of eggs in physiological saline. Monkeys were euthanized (sacrificed) by giving overdose of tranquilizer (chloral hydrate). “Permission under rules” was obtained for the purpose. Histopathological findings were observed in H&E-stained 5-6 *μ*m thick serial sections [10].

## 3. Results

### 3.1. Clinical Signs and Gross Lesions

**Figure 1:**
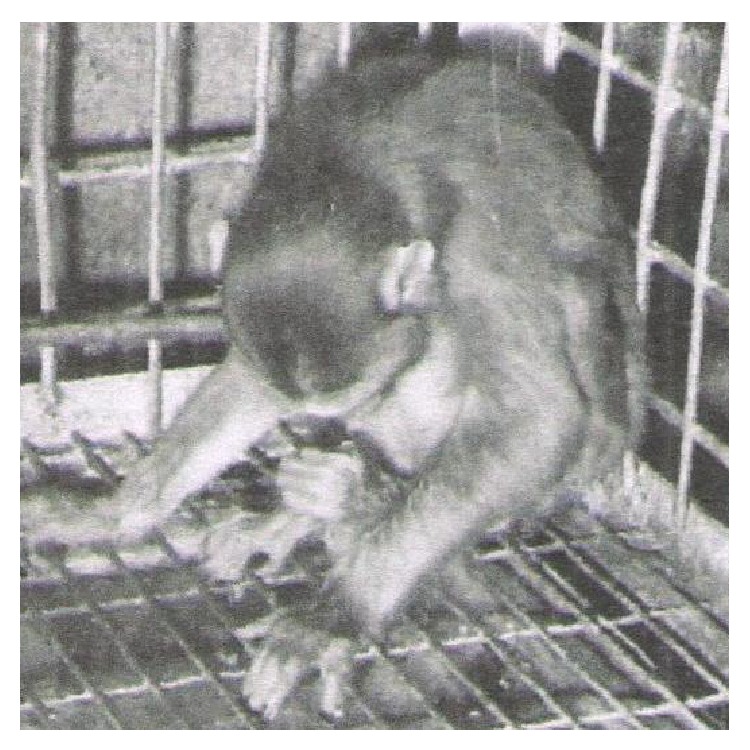
Photograph of monkey 46 DPI fed 12,000 eggs showing acute pressure syndrome and rapidly developing paralysis of hind quarter.

The heavily infected monkeys given 12,000 eggs each showed hyperexcitability at about 10 DPI, which lasted for 3–5 days. Between 15 and 45 DPI both monkeys appeared dull, developed progressive anorexia, preferred to sit quietly and, at times, were even found rolling. From 50 DPI, the monkeys developed acute dyspnoea, nervous compression, excitation syndrome, including epileptic seizures, muscular tremors, progressive paralysis of hind limbs (Figure 1), and digital cramps along with grinding of teeth, groaning, coughing, tendency to vomit, and excessive salivation. The animals thereafter showed profound weakness and failed to bear weight on the hind quarters, developed paralysis of both the hind and fore limbs, and, finally, died on 67 and 132 DPI, respectively.

**Figure 2:**
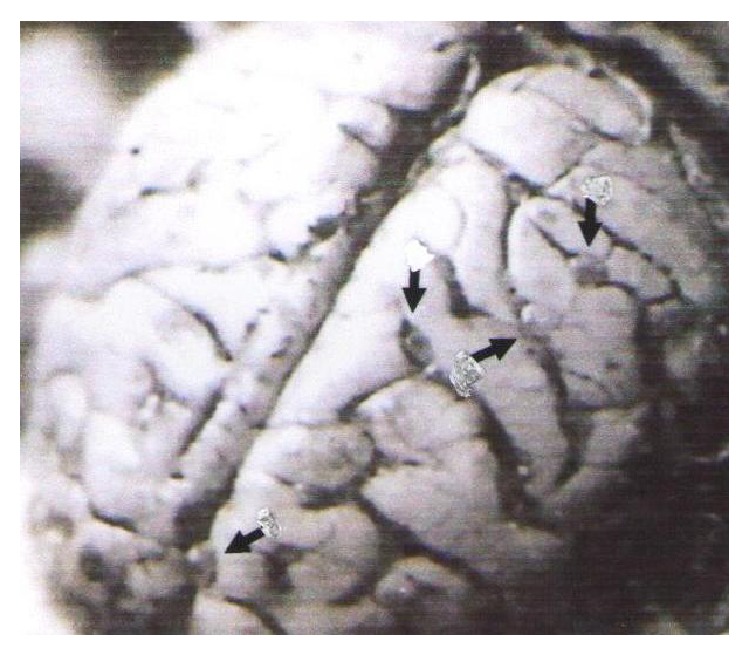
Photograph of the brain of a monkey given 12,000 eggs showing multiple cysts in the subarachnoid space.

The other two monkeys infected with 6,000 eggs each exhibited hyperexcitability between 10 and 12 DPI that improved considerably with time and had milder clinical signs, for example, anorexia, depression, grinding of teeth, and groaning from time to time. Both animals were necropsied at 144 and 147 DPI, respectively. On postmortem examination, all the infected monkeys showed numerous cysticerci in the brain (Figure 2), but none of the healthy control monkeys (necropsied simultaneously) had any cysticerci or gross lesion in the brain.

### 3.2. Histopathology

**Figure 3:**
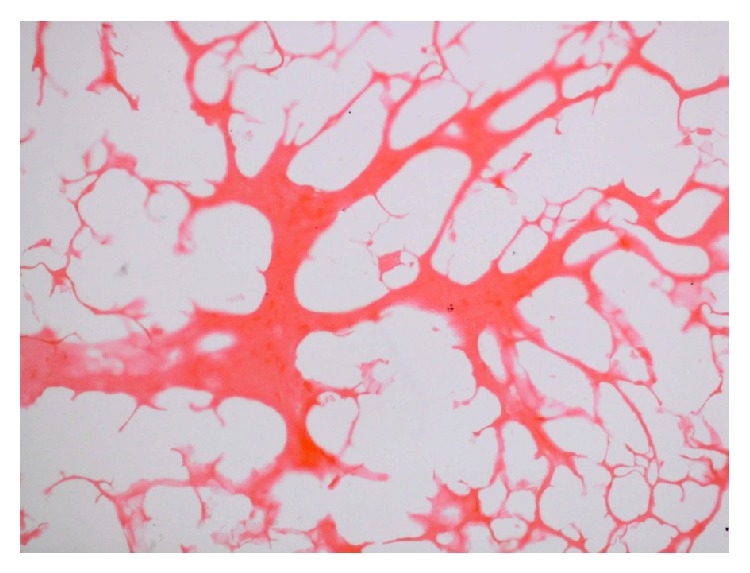
Marked liquefactive necrosis of neuropil with formation of variable sized cystic spaces lined by thin and atrophied parenchymal septa containing remnants of neuropil and chronic inflammatory cells, chiefly fibroblasts. H&E ×100.

The microscopic lesions varied from case to case. The monkeys that received a higher dose and died at 67 and 132 DPI showed marked liquefactive necrosis of the cerebrum with formation of variable sized irregular cystic cavities lined by atrophied parenchymal septa, containing remnants of neuropil (Figure 3) and chronic inflammatory cells, predominantly fibroblasts. It appeared that long term presence and the rapid growth of cysticerci led to chronic necrotizing inflammation, obstruction to the flow of cerebrospinal fluid, and/or retention of cystic fluid, thus building up intracerebral pressure. This might have resulted in massive neuronal deficit, culminating in clinical signs of nervous excitability or depression. The inflammation had extended from cerebrum to cerebellum producing widespread necrosis of Purkinje cells and thus resulting in staggering gait and balancing disturbances clinically.

**Figure 4:**
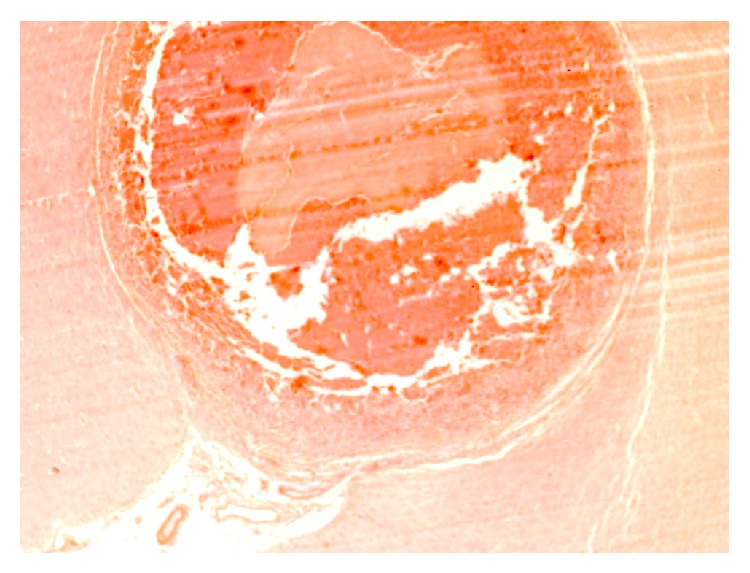
Foreign body granuloma in the cerebrum with chronic inflammation characterized by infiltration of macrophages, plasma cells, lymphocytes and eosinophils, large number of foreign body giant cells, and the central liquefactive necrosis, surrounded by a fibrous tissue capsule. H&E ×100.

**Figure 5:**
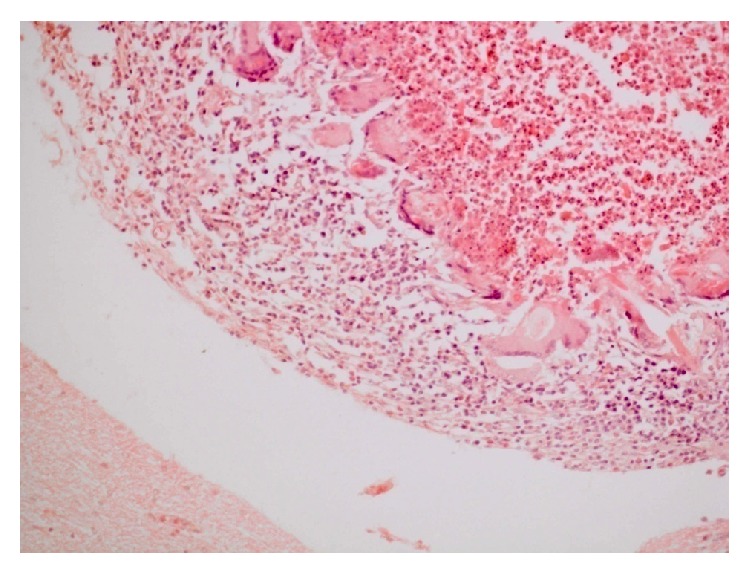
Higher magnification of previous figure showing inflammatory rim and foreign body giant cells more clearly. H&E ×200.

**Figure 6:**
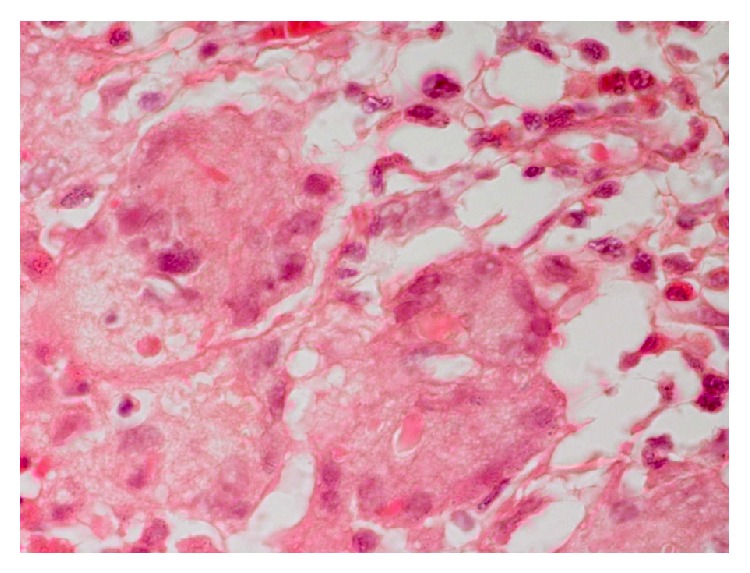
Even a higher magnification of the previous area shows foreign body of giant cells and chronic inflammatory cells. H&E ×400.

**Figure 7:**
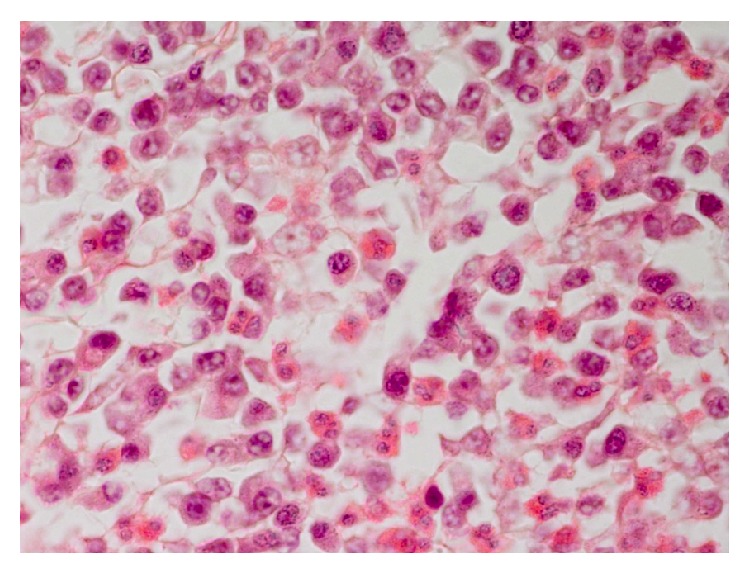
The inflammatory macrophages, a few lymphocytes, plasma cells and a few fibroblasts, and several eosinophils. H&E ×400.

**Figure 8:**
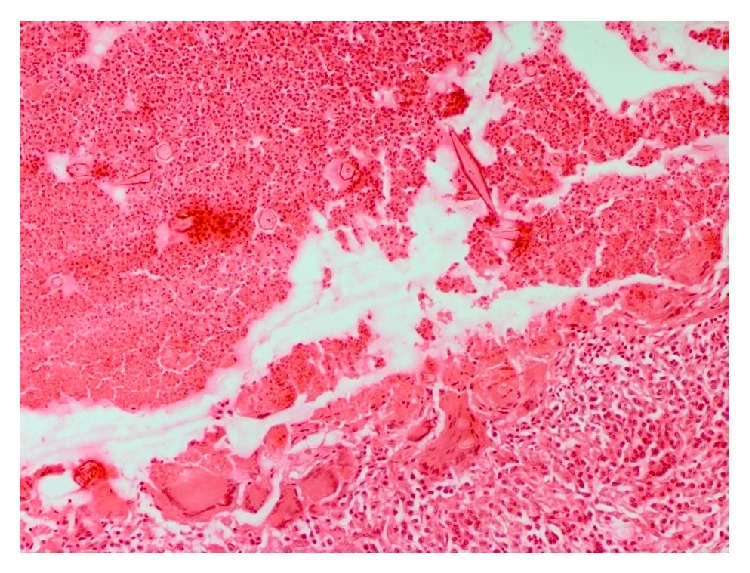
Foreign body granuloma in cerebrum encompassing the neural cysticercosis consisting of zone of inflammation, liquefactive/caseous necrosis, and cholesterol clefts besides a few small foci of foreign dystrophic calcification and cholesterol clefts. H&E ×200.

**Figure 9:**
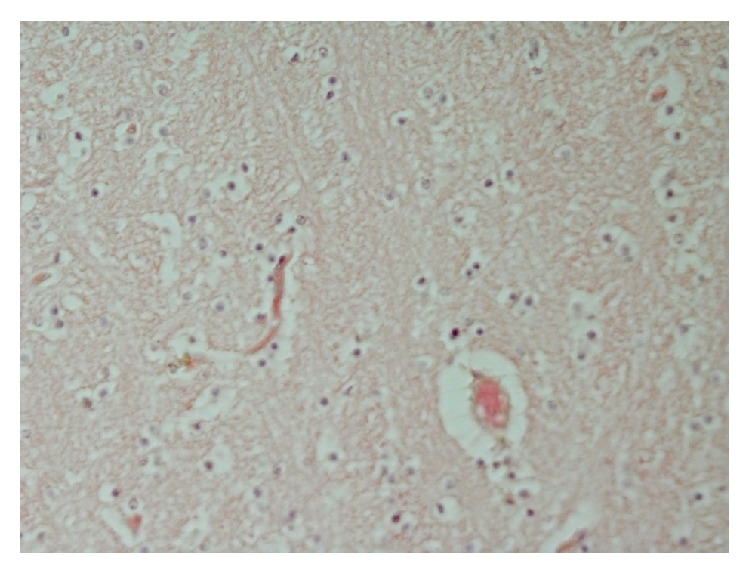
The cerebrum adjacent to the parasitic granuloma showing congestion, oedema, and mild perivascular cuffing. H&E ×200.

**Figure 10:**
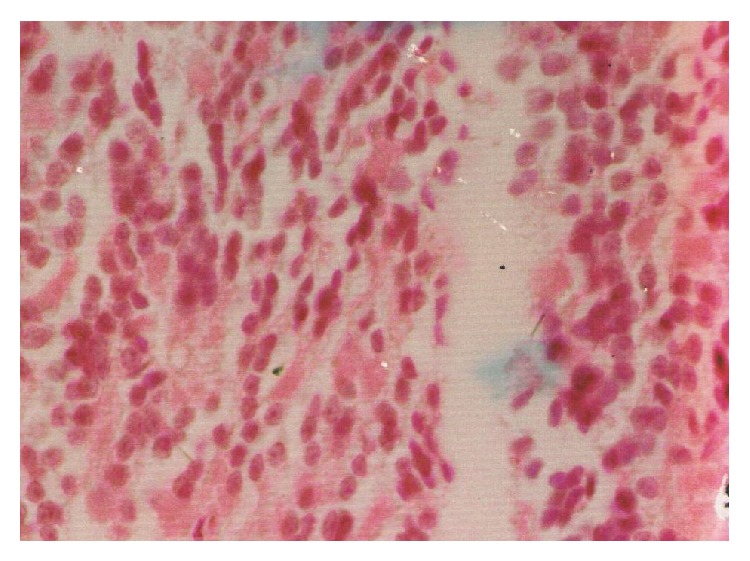
Photomicrograph of cerebellum showing marked distribution and atrophy of neuropil architecture. H&E ×100.

In the two monkeys that were given lower infective dose (6,000 eggs) each, there was formation of typical foreign body granulomas (Figures 4, 5, and 6) characterized by central liquefaction, surrounded by a rim of chronic inflammatory cells, comprising macrophages, plasma cells, lymphocytes, and eosinophils (Figure 7), besides large number of foreign body giant cells and fibroblasts (Figure 8). The necrosed areas at times contained foci of dystrophic calcification and cholesterol clefts (Figure 8). The areas of cerebrum adjoining the parasitic granulomas showed necrosis and atrophy of the parenchyma (Figure 9). Cerebellum showed marked distribution and atrophy of neuropil architecture (Figure 10).

## 4. Discussion

Neurocysticercosis may be asymptomatic or may show varied clinical manifestations. Clinical variations in neurocysticercosis are believed to be associated with critical anatomical location of the cysticerci and several other factors, namely, acute or chronic state of the illness, the number and severity of CNS lesions, species of the cysticerci (*Cysticercus cellulosae* or* C. racemosus*), the stage of development and involution of the cysts, and so forth [6]. The varied clinical signs in neurocysticercosis have also been ascribed to degeneration, absorption, cicatrization, calcification of cysts, and the type of immune and inflammatory responses [1, 11, 12]. In the present study, degeneration and liquefactive necrosis with formation of cystic spaces were noticed in the neuropil indicating neurologic deficit. However, typical cicatrization and dystrophic calcification were not recorded as reported earlier [6, 13].

Although neurocysticercosis is an important global zoonosis, responsible for increasing rate of seizures and epilepsy cases in humans, particularly in the underdeveloped and developing nations, little research effort has been targeted at understanding its pathogenesis. In experimental cysticercosis in pigs, Ohasi [7] reported definite epileptic seizures, convulsions, vertigo, and grinding of teeth. On the contrary, Herbert and Oberg [8] did not observe any clinical sign in piglets after 150 DPI of feeding them with 2,250–6,300 eggs each. Similarly, Hsieh [9] did not observe any neurological sign after experimentally feeding 1,00,000 eggs of* Taenia solium* in a Taiwan monkey (*Macaca cyclopis*). In the present experiment, hyperactivity in all the four infected monkeys was noticed between 10 and 15 DPI, that is, when oncospheres probably migrated, localized, and developed in the brain. At this stage, the clinical signs included convulsions, vertigo, grinding of teeth, respiratory sings, pressing the head against the cage, muscular crams and twitching, and acute pressure syndrome (Figure 1). Acute signs in heavily infected monkeys were observed from 40 DPI and exacerbated from 50 DPI onwards until death. In humans, it has also been speculated that when the parasite dies, the massive antigen is released with intensification of immune/inflammatory reactions that led to worsening of symptoms [6].

It is expected that the host exerts inflammatory and immune response against the cysticerci and in the process pathological lesions ensued including formation of granulomas. The granulomatous reactions in the brain in present study resembled broadly those seen in pigs and humans [5, 6]. However, the advantage of using monkeys instead of pigs, as models of human disease, in studying granulomas against cysticerci, lies in the fact that monkeys are phylogenetically closer to humans than pigs.

## 5. Conclusion

The diagnosis of neurocysticercosis currently relies heavily on brain imaging techniques and serology with granulomas in most cases detected through these methods. However, gross and histopathological correlates of the disease have hardly been studied. In this context, the current investigation reveals further insights into the development of clinical signs and associated pathology in neurocysticercosis. It also appeared from the present study that monkeys could serve as useful animal models for investigating human neurocysticercosis due to vast similarities in their cognitive responses and high susceptibility.

## References

[1] Arseni C., Cristescu A. (1972). Epilepsy due to cerebral cysticercosis. *Epilepsia*.

[2] Rajshekhar V., Joshi D. D., Doanh N. Q., van De N., Xiaonong Z. (2003). Taenia solium taeniosis/cysticercosis in Asia: epidemiology, impact and issues. *Acta Tropica*.

[3] Nash T. E., Mahanty S., Garcia H. H. (2013). Neurocysticercosis—more than a neglected disease. *PLoS Neglected Tropical Diseases*.

[4] de Aluja A. S., Vargas G. (1988). The histopathology of porcine cysticercosis. *Veterinary Parasitology*.

[5] Alvarez J. I., Londoo D. P., Alvarez A. L., Trujillo J., Jaramillo M. M., Restrepo B. I. (2002). Granuloma formation and parasite disintegration in porcine cysticercosis: comparison with human neurocysticercosis. *Journal of Comparative Pathology*.

[6] Pittella J. E. H. (1997). Neurocysticercosis. *Brain Pathology*.

[7] Ohasi M. (1931). Experimental studies on the *Cysticercus cellulosae*. *The Japanese Jounal of Veterinary Science*.

[8] Herbert I. V., Oberg C., Soulsby E. J. L. (1974). Cysticercosis in pigs due to infection with *Taenia solium* Linneus , 1758. *Parasitic Zoonoses—Clinical and Experimental Studies*.

[9] Hsieh H. C. (1960). Experimental transmission of *Cysticercus cellulosae* in Taiwan monkey, *Macaca cyclopis* (Swinhoe, 1862). *Formosan Science*.

[10] Bancroft J. D., Gamble M. (2008). *Theory and Practice of Histopathological Techniques*.

[11] Marquez-Monter H., Rojas R. A. M. (1971). Cysticercosis. *Pathology of Protozoan and Helminthic Diseases*.

[12] Bajpai H. S., Bhattacharya S. K. (1974). Epileptic fits in cysticercosis. *Tropical and Geographical Medicine*.

[13] Nash T. E., del Brutto O. H., Butman J. A., Corona T., Delgado-Escueta A., Duron R. M., Evans C. A. W., Gilman R. H., Gonzalez A. E., Loeb J. A., Medina M. T., Pietsch-Escueta S., Pretell E. J., Takayanagui O. M., Theodore W., Tsang V. C. W., Garcia H. H. (2004). Calcific neurocysticercosis and epileptogenesis. *Neurology*.

